# Human Bone Marrow Mesenchymal Stromal Cells Promote Bone Regeneration in a Xenogeneic Rabbit Model: A Preclinical Study

**DOI:** 10.1155/2018/7089484

**Published:** 2018-07-05

**Authors:** Juan Francisco Blanco, Jesús García-Briñon, Lorena Benito-Garzón, David Pescador, Sandra Muntión, Fermín Sánchez-Guijo

**Affiliations:** ^1^Department of Surgery, Orthopaedic Surgery and Traumatology, Faculty of Medicine, University of Salamanca, Salamanca, Spain; ^2^Institute of Biomedical Investigation of Salamanca (IBSAL), Hospital Universitario de Salamanca, Salamanca, Spain; ^3^Department of Cellular Biology and Pathology, Faculty of Medicine, University of Salamanca, Salamanca, Spain; ^4^Department of Hematology, Hospital Universitario de Salamanca, Salamanca, Spain

## Abstract

Significant research efforts have been undertaken during the last decades to treat musculoskeletal disorders and improve patient's mobility and quality of life. The goal is the return of function as quickly and completely as possible. Cellular therapy has been increasingly employed in this setting. The design of this study was focused on cell-based alternatives. The present study aimed at investigating the bone regeneration capacity of xenogeneic human bone marrow-derived mesenchymal stromal cell (hMSC) implantation with tricalcium phosphate (TCP) granules in an immunocompetent rabbit model of critical-size bone defects at the femoral condyles. Two experimental groups, TCP and hMSC + TCP, were compared. Combination of TCP and hMSC did not affect cell viability or osteogenic differentiation. We also observed significantly higher bone regeneration in vivo in the hMSC + TCP group, which also displayed better TCP osteointegration. Also, evidence of hMSC contribution to a better TCP osteointegration was noticed. Finally, no inflammatory reaction was detected, besides the xenotransplantation of human cells into an immunocompetent recipient. In summary, hMSC combined with TCP granules is a potential combination for bone regeneration purposes that provides better preclinical results compared to TCP alone.

## 1. Introduction

Despite the numerous advances in orthopaedic surgical techniques and new biomaterials, the repair of bone lesions continues to have a great room for improvement. Furthermore, the risk of bone diseases is far more prevalent due to aging. Bone fracture repairs have been intensively investigated at both clinical and basic level and still 5–10% of fractures resulted in either delayed or no repair [1].

The possibility of repairing an injured tissue by regeneration seems to be an attractive therapeutic option. Bone tissue remodelling process provides the capacity of self-regeneration after injury and the continual adaptation of bone mass and its architecture to the mechanical load [2]. Nevertheless, this regenerative capacity is limited to small defects. In clinical practice, with larger defects, often surgical intervention required the use of bone grafts for the treatment of different lesions, pseudoarthrosis, arthrodesis, and so on. Bone grafting frequency is indeed the second most frequent tissue transplantation worldwide, right after blood transfusion, used especially in oncologic surgery, traumatology, revision of prosthetic surgery, and spine surgery [3]. This is due to their easy use and handling, safety profile, cost and time advantages, and adaptability to a variety of clinical settings [4]. Common bone grafts include bone autografts, allografts, xenografts, and synthetic bone graft substitutes. Autologous bone continues to be the “gold standard” for grafting procedures, providing osteoinductive growth factors, osteogenic cells, and an osteoconductive scaffold [5]. However, limitations exist regarding donor site morbidity and graft availability. All other forms of bone repair have disadvantages compared to autograft. For instance, allograft has risk of disease transmission and synthetic graft substitutes lack osteoinductive or osteogenic properties [6]. The better understanding of bone repair biology has led to the development of new bone regeneration approaches through the use of synthetic grafts combining scaffolding properties with biological elements to stimulate cell proliferation and differentiation, and eventually osteogenesis [7]. The final objective is the full regeneration of the bone defect in the shortest possible time.

Calcium phosphates have been widely studied and used for bone repair [8, 9]. Because of their osteoconductive properties and their ability to integrate with bone tissue, most common synthetic bone graft substitutes involve hydroxyapatite (HA), *β*–tri-calcium phosphate (*β*-TCP), and their mixtures [10]. Nonporous, biological inert materials, such as ceramic and titanium, have been replaced by porous biomaterials, such as *β*-TCP, since they are resorbable and osteoconductive. A higher concentration of TCP in the bioceramic usually results in a higher resorbability [11, 12]. In the current study, Conduit™ TCP granules, a synthetic porous ceramic, was used as graft material. TCP is an osteoconductive material that allows the attachment of cells and the development of vascular networks.

Regarding combination of calcium phosphates with cellular components, bone marrow mesenchymal stromal cells (MSC) became well known at the end of the 1990s due to the evidence of being capable of multilineage differentiation. This property favored their use in bone tissue engineering, mostly in combination with an osteconductive scaffold as a graft material [13]. These cells could be obtained from different tissues, including bone marrow and adipose tissue [14]. MSC can be expanded and differentiated *in vitro* into cells with osteogenic phenotype [15, 16]. Their osteogenic differentiation could be guided through specific stimulus or signals such as growth factors [17, 18]. Besides their regenerative ability, MSC potential clinical applications have been boosted also due to their immunomodulatory capacity [19]. MSC exhibit immunomodulatory functions upon interaction with cells of both innate and adaptive immune systems [19, 20].

Previous studies have highlighted that autologous bone marrow stromal cells (MSC) are capable of regenerating bone defects when used in combination with bone substitutes [21–23]. Nevertheless, in our work, we have focused on the use of human MSC (hMSC) isolated from the iliac crest in combination with TCP.

The main purpose of the study was to probe the immunoprivileged properties of hMSC in a xenogeneic setting. We attempted to investigate the bone regeneration capacity of the xenograft in a critical-sized bone defect in an immunocompetent rabbit recipient. The challenge of the study was to get the viable addition of hMSC embedded in a common synthetic scaffold to promote bone regeneration in a xenogeneic model. A positive result could have a clinical relevance for any orthopaedic procedure requiring bone formation and may serve as preclinical basis to support the use of allogeneic cells.

## 2. Materials and Methods

### 2.1. Isolation and Growth of hMSC

Iliac crest bone marrow aspirates (5 ml) were obtained from patients that underwent spinal fusion for degenerative disc disease. They were otherwise healthy, and all of them were subjected to clinical and analytical evaluation to exclude the presence of relevant diseases and they were not receiving medical treatment for any condition, other than analgesics for the spinal degenerative disease. Median age of the donors was 60 years (range: 28–80 years), and male/female ratio was 1. Specimens were harvested according to the tenets of the Declaration of Helsinki and the Ethical Committee of the Hospital Universitario de Salamanca. All donors provided informed consent for the bone marrow sampling. A mononuclear fraction of bone marrow (CMN) was isolated by density-gradient centrifugation. Briefly, the bone marrow aspirate was diluted in Hank's balanced salt solution to increase the volume up to 12 ml. This cell solution was transferred to a centrifuge tube with 4 ml of Ficoll-Hypaque (Biochrom KG, Berlin, Germany) and was centrifuged at 1500 rpm for 30 min at room temperature. The interface cell layer was washed twice with Hanks 10 min at 1200 rpm at room temperature. The pellet was suspended with DMEM medium (Gibco BRL, Pailey, United Kingdom). A concentration of 10^6^ CMN/cm^2^ mononuclear isolated cells were seeded in a dish (T75 flaks) and cultured with DMEM supplemented with 10% fetal bovine serum (SBF; Bio Whittaker, Belgium) and antibiotics and incubated at 37°C with 5% CO_2_ in a humidified atmosphere. At 2-day intervals, the medium was replaced, and thus nonadherent cells were removed. Cells were allowed to expand up to reach around 70% of confluence. Then they were trypsinized and further subcultured at a density of 2.5 × 10^3^ cells/cm^2^. Cells were maintained until the 3rd passage, with a median of 11.48 ± 1.02 days in culture. At this stage, all the immunophenotypic analysis, the multilineage differentiation studies, and the remaining experiments were performed.

### 2.2. Flow Cytometric Analysis (FCA) of hMSC

Cell culture was characterized by flow cytometric analysis (FCA) for specific surface antigens, including CD105, CD73, CD90, CD34, CD45, CD14, CD19, and HLA-DR, in accordance with the international Society for Cellular Therapy (ISCT) recommendations [24]. Each sample analyzed by FCA contained 1 × 10^5^ cells. For data acquisition, a FACSCalibur flow cytometer (Becton Dickinson Biosciences, San Jose, CA, USA) was used.

### 2.3. Multilineage Differentation Potential of hMSC

For osteogenic differentiation, the hMSC were cultured with specific differentiation medium NH OsteoDiff Medium (Miltenyi Biotec, Germany). The hMSC culture was changed every 3 days during 10 days [25]. Afterwards, the monolayer was washed with PBS (phosphate-buffered saline), cooled 70% ethanol solution fixed for 10 min at room temperature, and then incubated for 30 min with 5-bromo-4-chloro-3-indolyl phosphate/nitro blue tetrazolium (BCIP-NBT, Sigma, B5655). For a better contrast, an incubation in 1 ml of hematoxylin for 2 min was done. Then, the monolayer was washed with distilled water and observed under an optical inverted microscope (Olympus BX41).

For adipogenic differentiation, the hMSCs were cultured with differentation medium NH AdipoDiff Medium (Miltenyi Biotec, Germany). The hMSC culture was changed every 3 days for 21 days. Afterwards, the monolayer was washed with PBS, 10% formalin fixed for 2 min at room temperature, and then incubated for 1 hour with 1 ml Oil Red O solution (Merk, Darmstadt, Germany) at room temperature.

### 2.4. Material

A synthetic porous ceramic graft material composed of tricalcium phosphate, commercially available as Conduit TCP (DePuy Orthopaedics Inc.) was employed as scaffold either alone or in combination with the hMSC. Conduit TCP consists of irregular granules with interconnected porosity of about 70% and pores of 1–600 *μ*m in diameter.

### 2.5. Animals and Surgical Procedures

All animal handling and surgical procedures were conducted according to the European Community guidelines for the care and use of laboratory animals (Directive 2010/63/EU) and approved also by the local ethical committee of the University of Salamanca, in accordance with Spanish law (RD 53/2013).

**Figure 1:**
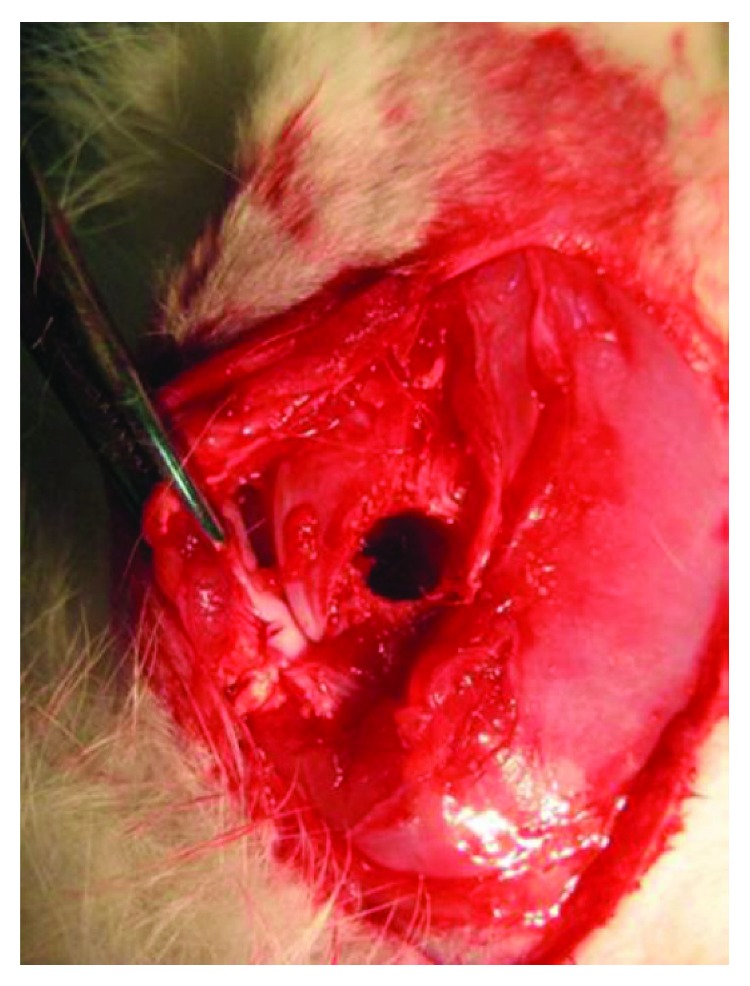
Critical size femoral defect of 6 mm of diameter in a rabbit model.

Fourteen immunocompetent mature male New Zealand rabbits weighing between 3.0 ± 0.5 kg were injected intramuscular an anaesthesia mixed of Xylacine 5 mg/kg (Rompun® 2%, 25 ml) and Ketamine 35 mg/kg (Ketolar® 50 mg/ml). Anesthetized sate was maintained with isoflurane and oxygen ventilation. Once each animal was anesthetized, the knees were disinfected with 4% chlorhexidine and shaved. Once the femoral condyle was exposed, an established bone critical-size defect [26–30] was created in the trabecular bone of the lateral area of the femoral condyles, close to the cartilage (Figure 1). A cylindrical hole with a diameter of 6 mm and depth of 10 mm was drilled under continuous cooling with saline using an electric motor.The right femur defect was filled with TCP granules loaded with ex vivo expanded hMSC. The left femur defect was filled with TCP only, serving as control group. The rabbits were allowed to walk 2-3 hours after the surgery and were kept individually in large cages. After 12 weeks of implantation, animals were sacrificed by means of intravenous injection with pentobarbital (120 mg/kg) (Penta-Hypnol®) following general anaesthetic. Bone samples were harvested for histological study.

### 2.6. Histological Study

The rabbit condyles were fixed in 10% neutral buffered formalin. The regions containing the defects were dehydrated in graded series of alcohol/water mixture followed by complete dehydration in absolute alcohol. Afterwards, the specimens were embedded in poly-methylmethacrylate resin and cut into 5–7 *μ*m thick sections on a microtome. Sections were desplastified and rehydrated prior to staining with toluidine blue.

#### 2.6.1. Evaluation of Bone Regeneration

Assessment of bone regeneration was performed following a modification of the histological evaluation method from Lucaciu et al. [31] adapted for our study. Histological examination of slices was accomplished on a Zeiss Axio Scope A.1 photomicroscope. Representative sections obtained from each of all the animals were examined, and different fields, which include the lesion region, were photographed by using the ×10, ×20, and x40 objectives. The images were then analyzed and evaluated according to 10 parameters (see Table 1). By adding up the score given to each parameter, we obtained the histological score for each subject individually, which represents the sum of all evaluated parameters, with the maximum potential value being 28.

### 2.7. Immunofluorescence

To identify the presence and participation of the hMSC in bone regeneration, immunofluorescence detection of a glucosylated protein present in the human mitochondrial membrane was detected by using a mouse monoclonal antibody (MAB1273 Millipore). Samples were analyzed and photographed under a photomicroscope (Zeiss Scope A1) equipped with epifluorescence and appropriate filter sets.

### 2.8. Data Analysis

The histopathological results were scored in a double-blinded manner, and the figures presented in the manuscript are representative images.

Statistical analysis of the data was performed with the IBM SPSS (v. 23.0) application. Normal distribution of data from both, control and experimental groups, was tested with Shapiro-Wilk test (recommended for samples with *n* < 50). In order to detect statistically significant differences between TCP and hMSC-TCP groups, we applied the *T-*test for related samples (since control and experimental femoral condyles belonged to the same animal). The significance threshold was set at *p* < 0.05.

## 3. Results

### 3.1. Isolation and Characterization of hMSC

**Figure 2:**
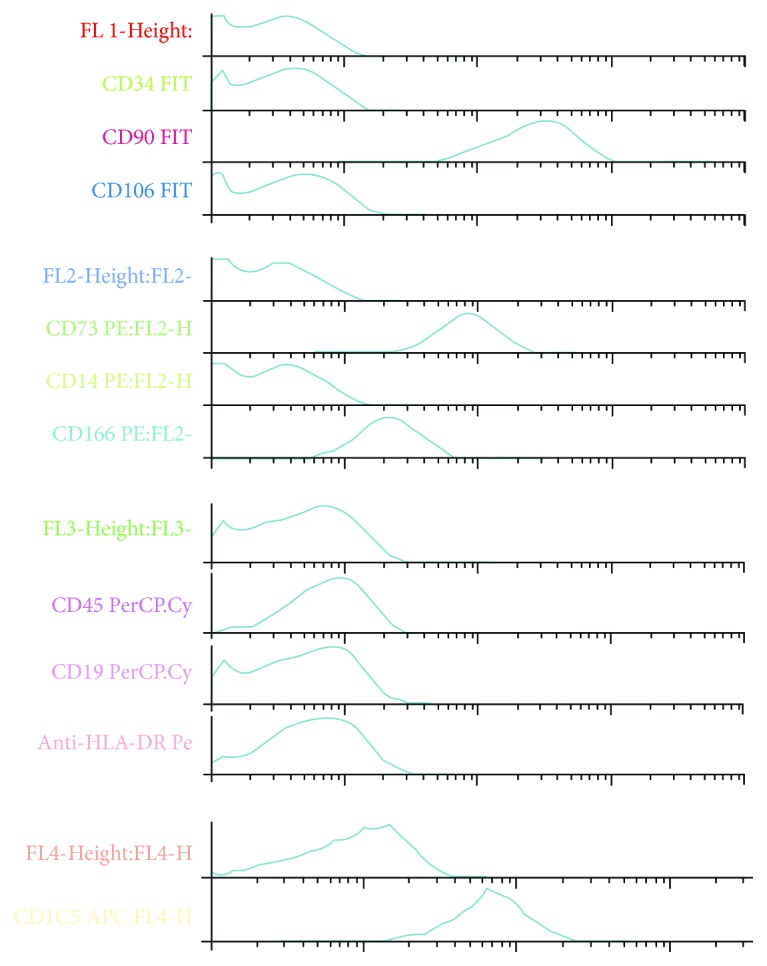
Immunophenotypic profile by flow cytometry.

In all cases, hMSC were isolated and expanded in vitro and acquired the characteristic spindle-shape morphology. As indicated in the methods, cells were grown up to third passage and median time for each passage was 11.48 ± 1.02 days. After the flow cytometric analysis, the characteristic immunophenotypic profile was demonstrated. Cells were positive for CD90, CD73, and CD105 and negative for CD45, CD34, CD14, CD19, and HLA-DR (Figure 2).

**Figure 3:**
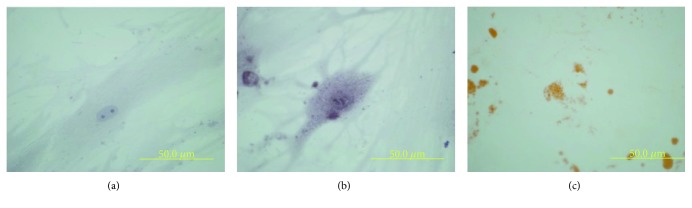
In vitro multilineage differentiation of hMSC: (a) control, (b) osteoblastic differentiation, and (c) adipocytic differentiation (scale bar: 50 *μ*m).

In addition, multilineage differentiation into osteblasts and adipocytes was demonstrated by BCIP-NBT and Oil-Red-O staining, respectively (Figure 3).

### 3.2. Clinical Observations

There were no complications either during the surgical procedure, the postoperative course, or the bone biopsies performed 12 weeks after surgery. Only two of the cases presented infection signs in defects treated only with TCP, so these were discarded for the analysis.

### 3.3. Histological Analysis

**Figure 4:**
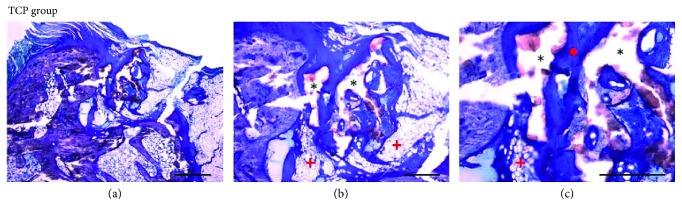
Photomicrographs of histological sections stained with toluidine blue taken from the TCP group (a–c). (a) The defect area was still evident 12 weeks after surgery. Connective tissue and adipose bone marrow were generated at the injured area (scale bar: 1000 *μ*m). (b) The remaining TCP material was not fully osteointegrated (^∗^) (scale bar: 500 *μ*m). (c) Scarce trabecular bone formation in association with TCP granules. Nonosteointegrated TCP granules (^∗^) were surrounded by connective tissue (red circle) or adipose bone marrow (red plus sign) (scale bar: 500 *μ*m).

**Figure 5:**
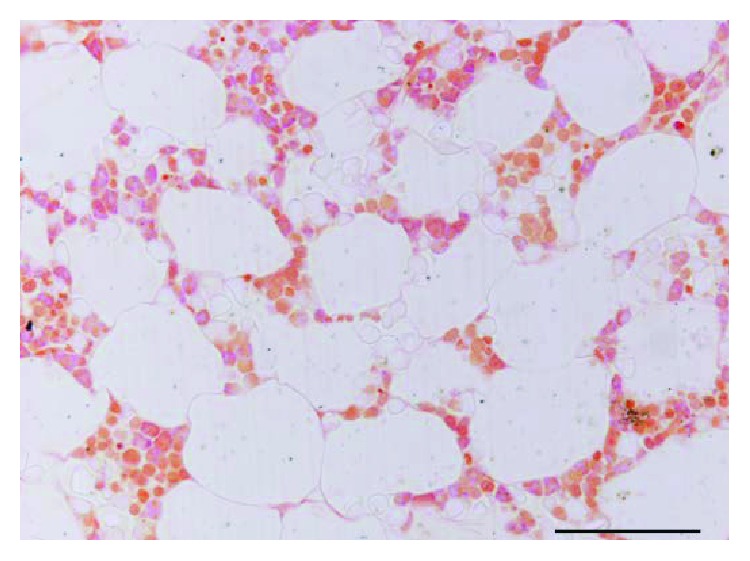
Photomicrograph of histological section from the TCP group. Evidence of empty spaces filled by adipose bone marrow due to the absence of osteointegration of TCP granules (scale bar: 150 *μ*m).

Histology sections were blindly examined by bright-field microscopy. All samples showed signs of bone regeneration, in a greater or lesser extent, and no infiltration by inflammatory cells was found. However, bone formation was different between the two groups: TCP and hMSC-TCP. In particular, toluidine blue-stained sections from the TCP-only group revealed that the defect was still evident (Figure 4(a)). The bone defects were not regenerated in any case, and TCP granules were observed at the injured region without any evidence of osteointegration (Figures 4(a) and 4(b)). Although some TCP granules could be observed at the surface of the bone, the tendency was to find granules not osteointegrated, so they were surrounded by loose connective tissue or adipose bone marrow (Figure 4(c)). Indeed, most of the nonosteointegrated TCP granules were washed out during the histological processing, resulting in the observation of empty spaces upon examination by light microscopy (Figure 5). Nevertheless, evidence of a light bone formation within the defect area was observed (Figure 4(b)) with scarce trabecular bone formation in association with TCP granules although the generated bone was not enough to fully regenerate the defect (Figures 4(b) and 4(c)). Mainly connective tissue and adipose bone marrow were observed at the injured area (Figures 4(a) and 5).

**Figure 6:**
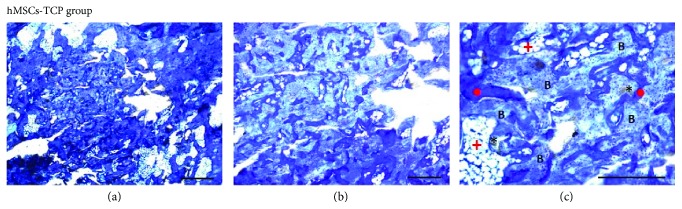
Photomicrographs of histological sections stained with toluidine blue taken from the hMSC-TCP group (a–c). (a) Almost complete bone defect regeneration (scale bar: 1000 *μ*m). (b) Newly formed bone showed disorganized and anastomosed trabeculae. TCP granules were osteointegrated (scale bar: 500 *μ*m). (c) TCP granules were surrounded by newly formed trabecular bone (**B**). Not completely osteointegrated TCP granules (^∗^) were in contact with adipose bone marrow (red plus sign) or connective tissue (red circle) at their bone-free surface. Normal osteocytes were present at the regenerated trabecular bone (scale bar: 500 *μ*m).

Concerning the sections from the hMSC-TCP group, they showed an almost complete regeneration of the bone defect (Figure 6(a)). In these cases, new trabecular bone was formed and osteointegrated TCP particles could be detected among the newly regenerated bone (Figures 6(b) and 6(c)). TCP granules were usually found adjacent to or embedded within the newly formed bone, without interposition of connective tissue (Figure 6(b)). Areas of TCP granules not covered by bone were in direct contact with adipose bone marrow (Figure 6(c)). Newly formed trabecular bone presented the typical cellular component, with the presence of osteocytes among the newly mineralized bone matrix (Figure 6(c)).

**Figure 7:**
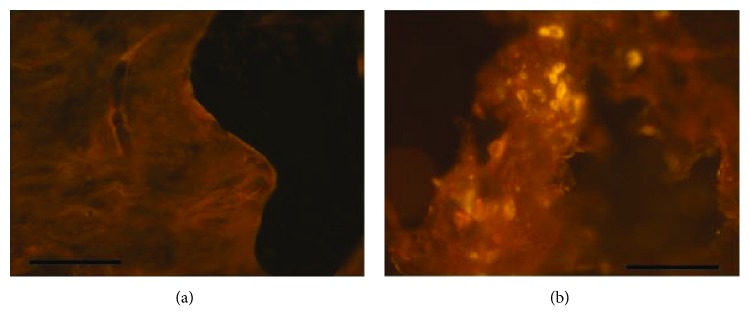
Fluorescent images of the TCP group (a) and the hMSC-TCP group (b) (scale bar: 150 *μ*m). (a) No immunofluorescence was detected. (b) Specific immunofluorescence detection of survival hMSC at the critical-size bone defect in inmunocompetent rabbit 12 weeks after surgery.

To identify and confirm that the implanted hMSC had survived and contributed to the regeneration process, the immunofluorescence detection of human mitochondrial antigen was performed (Figure 7). As expected, no signal was detected in the sections from the TCP-only group (Figure 7(a)). By contrast, evidence of immunofluorescent cells intermingled between the matrix of the newly formed bone was easily observed in the hMSC-TCP group (Figure 7(b)). These findings strongly suggest that hMSC had survived for 12 weeks in the inmunocompetent rabbit model.

### 3.4. Histological Score

After 12 weeks of implantation, in the TCP group, there was very scarce bone formation and it was mostly located at the periphery or at the surface, whereas in most of the hMSC-TCP-treated animals, bone formation was observed both in the central and in the peripheral regions of the lesion. Osteoblasts, osteocytes, and osteoclasts were scarcely observed in the TCP group, but they were abundantly observed in all regions of the injured tissue of the hMSC-treated femoral condyles. Moreover, in the hMSC-TCP group, the osteoclastic degradation of the scaffold and its replacement with mature bone was abundant in all regions of the defect, which was almost completely filled with mature bone tissue. These features were very rarely observed in the TCP-treated femoral condyles.

**Figure 8:**
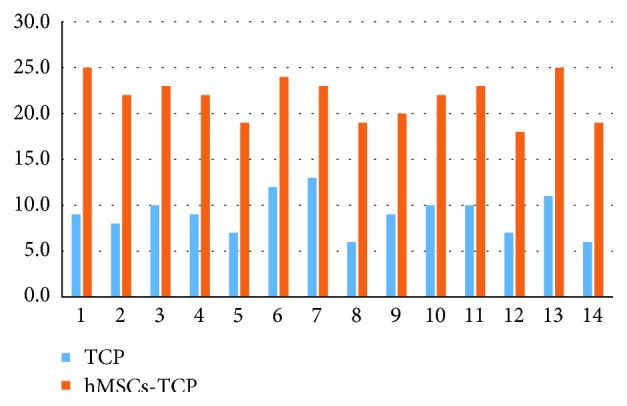
Representation of score values (vertical axis) of the subjects included in the study (horizontal axis). Score values for bone regeneration of TCP group (left femur) were clearly lower than those of the hMSC-TCP group (right femur).

As already indicated, these qualitative histological observations were scored according to the parameters included in Table 1, and the obtained values, for each of the 14 analyzed subjects, are represented in Table 2 and Figure 8. The mean histological score of the TCP-only group was significantly lower (9.07 ± 0.57) compared to the hMSC-TCP group was 21.71 ± 0.62; *p* < 0.001).

## 4. Discussion

The main aim of the current work was to ascertain if human MSC displayed their immunoprivileged properties in a xenogeneic setting of bone defect. In addition, we planned to compare in this setting the therapeutic effect of hMSC combined with a TCP-based carrier in a well-established model for critical-size defect. Interestingly, we have observed both the absence of inflammatory reaction in the implant area and a significantly higher bone regeneration ability of the hMSC-TCP group compared to the TCP-only group. These results may support the potential role of this combination in a clinical trial using allogeneic cells.

The rabbit model we have used for critical-sized defect of bone healing has been extensively used [32, 33]. We have observed that the largest part of the center of the defect remained not regenerated, and only a small amount of bone formation was observed at the margins. Rabbits are commonly used as animal models in approximately 35% of the musculoskeletal researches in medical investigation [34]. This is in part due to ease of handling and size.

The human body has an extensive capacity to regenerate bone tissue after trauma. However, large defects cannot be restored without intervention and often lead to nonunion. Due to the multiple limitations associated with the use of autografts or bank-stored bones for bone reconstruction, investigators have developed alternative solutions. Recent tissue engineering approaches have attempted to create new bone based on seed MSC onto calcium phosphate ceramic scaffolds. Hydroxyapatite- (HA-) based ceramics presented slow resorption rate producing bone ingrowth onto a porous surface rather than a true bone regeneration [15, 35, 36]. Nevertheless, synthetic porous *β*-TCP granules, such as Conduit TCP, possess a fast resorption rate and osteoconductivity. Due to calcium phosphate ceramics' poor mechanical properties, their success depends on their capacity of reabsorbability and degradation while promoting bone regeneration [37, 38]. For this reason, in this work, we have used TCP granules, due to its better reabsorption compared to HA. To allow bone replacement, a gradual degradation let the material first, serving as a scaffold for bone formation, and then permitting replacement of the material with bone. The main objective was to promote bone formation de novo, repairing instead of replacing the bone scaffold. The higher bone regeneration process is related to the better properties of the resulting tissue. According to the results of our work, histological study demonstrated that the hMSC-TCP group could effectively produce an almost complete regeneration of bone defect *in vivo* in comparison with TCP group and these results were statistically significant using a histological scoring system. Besides, the hMSC-TCP group showed better osteointegration of TCP granules in comparison to the TCP group, resulting in a more stable tissue.

Cell-based therapies are already used in musculoskeletal pathologies, such as bone fracture, pseduoarthrosis, and osteochondral defects [39, 40]. MSC seeding onto natural or synthetic biomaterials represents the most effective way to induce regeneration and repair of bone and cartilage [41]. Studies have been shown very effective approach to repair bone defects when local implantation of porous biomaterials covered with autologous bone marrow MSC has been tested in large bone defects [42]. Scaffolds for MSC, regardless of the material from which they are formed, should encourage MSC adhesion, proliferation, and differentiation to elicit bone formation. Pioneering studies showed that pore sizes less than 15–50 mm result in fibrovascular ingrowth, pore sizes of 50–150 mm encourage osteoid formation, and pore sizes greater than 150 mm encourage the ingrowth of mineralized bone [22]. In the case of Conduit TCP, it presents an average pore size of 1–600 *μ*m which optimizes cellular in-growth and attachment. The connected porosity allows for developing a vascular network. It can be hypothesized that these properties of the scaffold may favor the osteogenic differentiation ability of the MSC, and this may favor in addition the secretion of growth factors that may contribute to bone repair in a number of diseases where bone degeneration is a key factor and that aging MSC is susceptible to improve their function by the interaction with this donor MSC-derived growth factors.

Besides, it must take into account that MSC possess strong immune regulatory properties that are present in cells from different animal species, although with variable and only partially clarified mechanisms. MSC may suppress immune reactions *in vitro* and *in vivo* in a major histocompatibility complex- (MHC-) independent manner [43, 44]. Previous studies have indicated that both undifferentiated bone marrow-derived stem cells (BMSC) and adipose-derived stem cells (ASCs) exhibit immunosuppression and immunoprivilege properties [45, 46].

It has been reported that BMSC may be immune-privileged cells that do not elicit immune responses due to an absence of immunologically relevant cell surface markers. In addition, BMSC have immunomodulatory function. For that reason, BMSC theoretically can make them impervious to immunorejection following xenogeneic transplantation. Previous studies have reported opposite results ranging from no survival to differentiation into destination cells [47–49].

There are many works where xenogeneic MSC was transplanted into immunosuppressed animal models [50]. Based on this immunoprivilege situation, our work was based on a xenotransplant using an immunocompetent animal model. It is suspected that MSCs are not immunogenic even under xenogeneic conditions but still there is no a clear evidence if MSCs could be applied in immunocompetent animal models. In other studies, hMSC survived after transplantation in the spinal cord [51], intervertebrate discs [52], eye [53], or heart [54] in nonimmunosuppressed rats. Previous work about xenotransplant of human cord blood-derived stem cells to achieve bone regeneration found surviving cells until 4 weeks in immunocompetent rats [55], but with longer time, the human cells were eliminated by the host organism. For that reason, if more experimentation time was needed, animals would be immunosuppressed. In our work, there is evidence that hMSC survive within the bone defect up to 12 weeks in an immunocompetent rabbit model.

To our knowledge, this is the first demonstration of the survival of transplanted xenogeneic hMSC in a femur condyle defect and the bone formation without immune suppression. We observed that hMSC in combination with TCP granules successfully promotes bone formation in a critical-sized bone defect and the bone regeneration capacity was greater in comparison with TCP granules alone, where less bone regeneration process occurred. In the hMSC-TCP group, although bone regeneration of the defect was not complete, the presence of viable hMSC capable of osteogenesis was evident at 12 weeks in an immunocompetent rabbit model. Consistent with previous studies, hMSC combination with a scaffold resulted in significant bone formation when compared with scaffold only [55–57].

The results of the current study should be adequately interpreted taking into account some of the limitations of our work that include the small number of animals used and that the animals were analyzed histologically in a single time-point (after 12 weeks of implant).

In summary, our findings indicated that xenogeneic transplantation of hMSC using a calcium phosphate osteoconductive material promotes almost a complete regeneration of critical-size bone defect in an immunocompetent rabbit model. TCP granules can support proliferation and viability of hMSC. The incorporation of hMSC to TCP improves its osteointegration and bone regeneration. These results support the use of this combination in a nonautologous setting that should be explored in clinical trials.

## Figures and Tables

**Table 1:** Histological evaluation record.

Histological score
*(1) Bone formation* 0: absent 1: present at the periphery 2: present centrally 3: present centrally and at the periphery

*(2) Bone formation* 0: absent 1: present at the surface of the graft 2: present in the depth of the graft

*(3) Osteoblasts* 0: absent 1: present at the periphery 2: present centrally 3: present centrally and at the periphery

*(4) Osteocytes* 0: absent 1: present at the periphery 2: present centrally 3: present centrally and at the periphery

*(5) Osteoclasts* 0: absent 1: present at the periphery 2: present centrally 3: present centrally and at the periphery

*(6) Immature bone* 0: present centrally 1: present at the periphery 2: absent

*(7) Mature bone* 0: absent 1: present at the periphery 2: present centrally 3: present centrally and at the periphery

*(8) Osteoclastic degradation of the scaffold* 0: absent 1: present at the periphery 2: present centrally 3: present centrally and at the periphery

*(9) Scaffold replacement with mature bone* 0: absent 1: present at the periphery 2: present centrally 3: present centrally and at the periphery

*(10) Bone tissue* 0: absent 1: present at the periphery 2: present centrally 3: present centrally and at the periphery

**Table 2:** Score of the subjects included in the study according to the evaluated histological parameters included in Table 1.

Subject number	TCP (left femur)	hMSC-TCP (right femur)
1	9	25
2	8	22
3	10	23
4	9	22
5	7	19
6	12	24
7	13	23
8	6	19
9	9	20
10	10	22
11	10	23
12	7	18
13	11	25
14	6	19
Mean ± S.E.M.	9.07 ± 0.57	21.71 ± 0.62

## Data Availability

The data used to support the findings of this study are available from the corresponding author upon request.
